# Supplementary figures and images for: Capsule type defines the capability of Klebsiella pneumoniae in evading Kupffer cell capture in the liver
Source: PLoS Pathog. 2022 Aug 1;18(8):e1010693. doi: 10.1371/journal.ppat.1010693 (PMC9342791; doi:10.1371/journal.ppat.1010693)

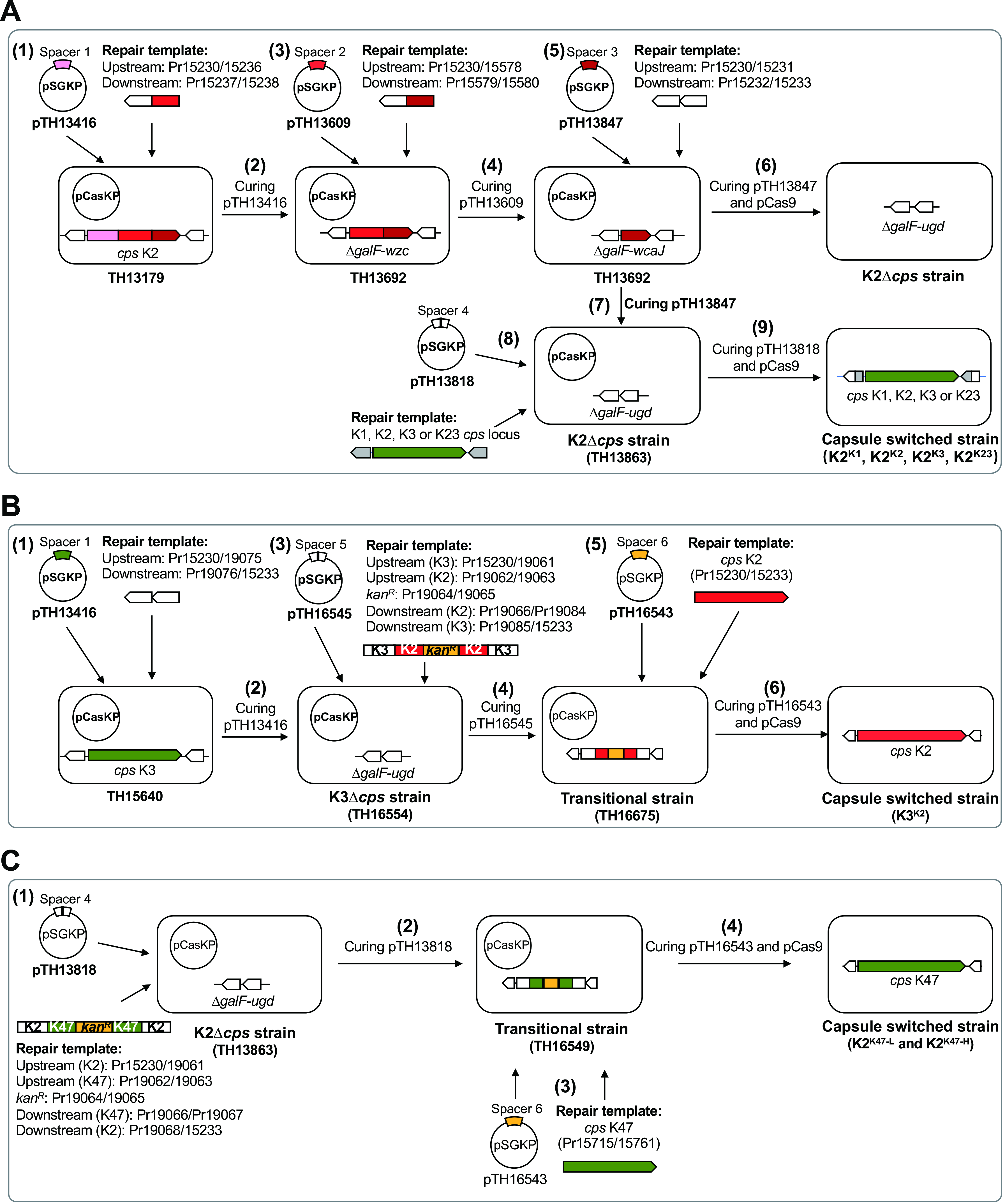

Supplement: S1 Fig — (A) Construction of capsule isogenic variants K2K1, K2K2, K2K3 and K2K23. The cps cluster of ATCC43816 was divided into three segments indicated as the pink, red and crimson regions to delete sequentially in the same manner (step 1–6). (1) Deletion of the first segment of cps cluster. The spacer targeting cps cluster was cloned into sgRNA-expressed plasmid pSGKP-spe. The repair template for repairing Cas9-cleaved DNA by homologous recombination was constructed by fusion PCR with up- and downstream sequence of target region. Spacer-introduced plasmid and repair template were co-electroporated into Cas9-expressed strain generating strain with deletion of cps genes (ΔgalF-wzc). (2) Curing the spacer-introduced plasmid. (3–7) Deleting the second segment (3 and 4) and third segment (5–7) of cps cluster generated Δcps strain by the same procedure as in (1) and (2). (8–9) The cps cluster of other capsule types was transformed into the Δcps strain to yield capsule replacement strain. The spacer-introduced plasmid expressing sgRNA to target junction of cps up- and downstream region and donor cps amplicons from 12908 (K1), ATCC43816 (K2), TH12849 (K3) or TH12852 (K23) strain were co-transformed to Δcps strain generating capsule-switched strain. (B) Construction of capsule isogenic variants (K3K3, K3K2, K7K7 and K7K2) using LV recipient strains. (1–2) Deletion of the cps cluster. (3–4) Introduction of the 5’ and 3’ K2 homologous arms (1 kb each) and kanamycin resistance gene (kanR) as screening marker to construct the transitional strain. (5–6) The K2 cps cluster of ATCC43816 was transformed into the transitional strain to yield capsule replacement strain K3K2. The K7K7 were constructed in the same way. (C) Construction of capsule isogenic variants K2K47-L and K2K47-H. (1–2) Construction of the transitional strain with K47 homologous arms and kanR gene. (3–4) The cps cluster amplicons of TH 12845 (K47, high virulence) and TH 12846 (K47, low virulence) capsule types was transfo [file ppat.1010693.s001.tif]

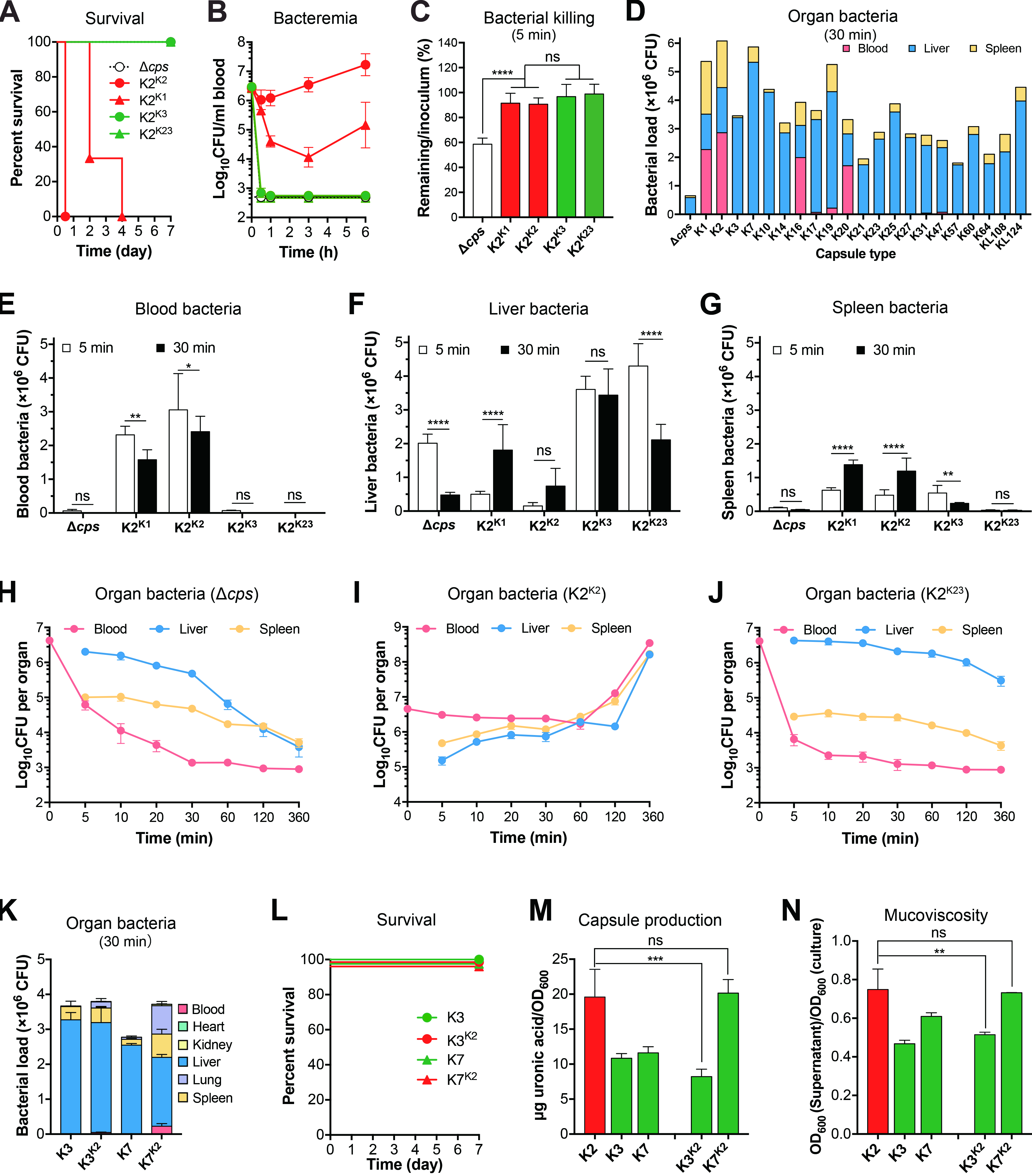

Supplement: S2 Fig — (related to Fig 3). Survival rate (A) and bacteremia kinetics in 6 hr (B) of the CD1 mice i.v. infected with 5 × 10^6^ CFU of capsule isogenic variants derivatives of ATCC43816. n = 6. Percentage of total viable bacteria at 5 min post infection to the initial inoculation (C). The total bacteria burdens of blood and five major organs divided by the initial inoculum are presented as percentage of inoculum. n = 6. Viable bacteria of wildtype K. pneumoniae strains representing 21 capsule types in organs and blood at 30 min (D). n = 1. Changes of bacterial load in blood (D), liver (E) and spleen (F) from 5 to 30 min post infection. n = 6. Viable bacteria of Δcps (G), K2K2 (H) or K2K23 (I) in blood, liver and spleen within 6 hr after infection. n = 6. Bacterial load in blood and organs (K) and survival rate (L) of mice infected with capsule-switched derivatives of LV strain at 30 min. n = 4–6. Capsule production (M) and mucoviscosity (N) of capsule hybrid strains (K3K2 and K7K2), the related LV recipients (K3 and K7) and HV donor (K2). The data are presented as mean ± SD. One-way ANOVA with Tukey’s (C) or Dunnett’s (M and N) multiple comparisons test, and two-way ANOVA with Sidak’s (E-G) multiple comparisons test were performed. ns, not significant; *, P < 0.1; **, P <0.01; ***, P <0.01; ****, P <0.0001. (TIF) [file ppat.1010693.s002.tif]

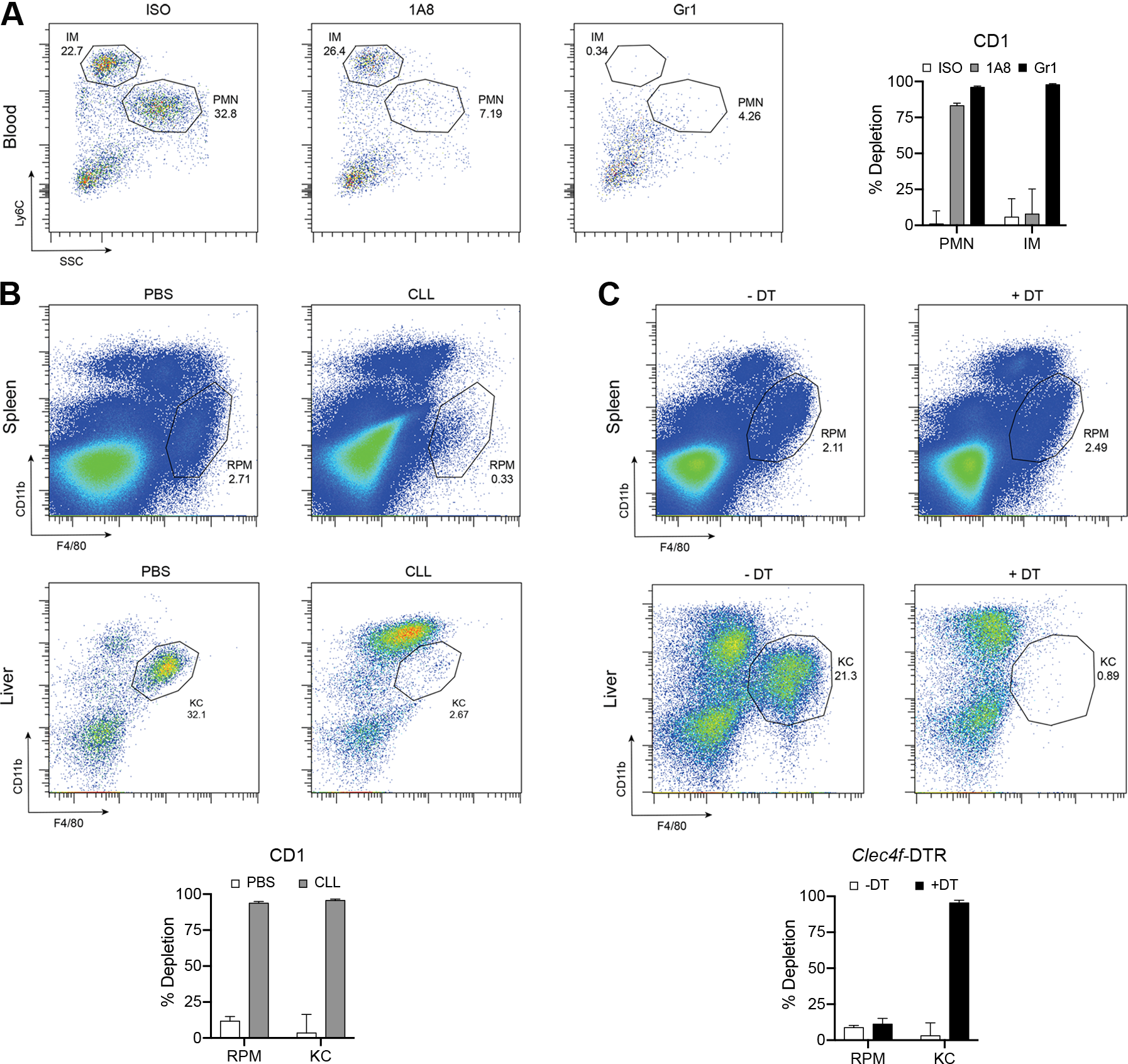

Supplement: S3 Fig — Depletion efficiency of neutrophil and monocyte (A). Mice were treated with ISO (isotype control), 1A8 antibody (depleting neutrophil) or Gr1 antibody (depleting neutrophil and inflammatory monocyte). The proportion of neutrophils (Ly6Clow/SSChigh) and inflammatory monocytes (Ly6Chigh/SSClow) in blood myeloid population (CD45+ / CD11b+) was measured. CD1, n = 2. Depletion efficiency of tissue resident macrophage in spleen and liver (B, C). The ratios of RPM and KC (CD11blow/F4/80+) in the immune cells (CD45+) of CD1 mice treated with PBS or CLL (depleting macrophages) for 72 hr (B) and Clec4f-DTR mice treated with or without DT (specific depleting KC) for 12 hr (C) were measured. n = 2. The data are presented as mean ± SD. (TIF) [file ppat.1010693.s003.tif]

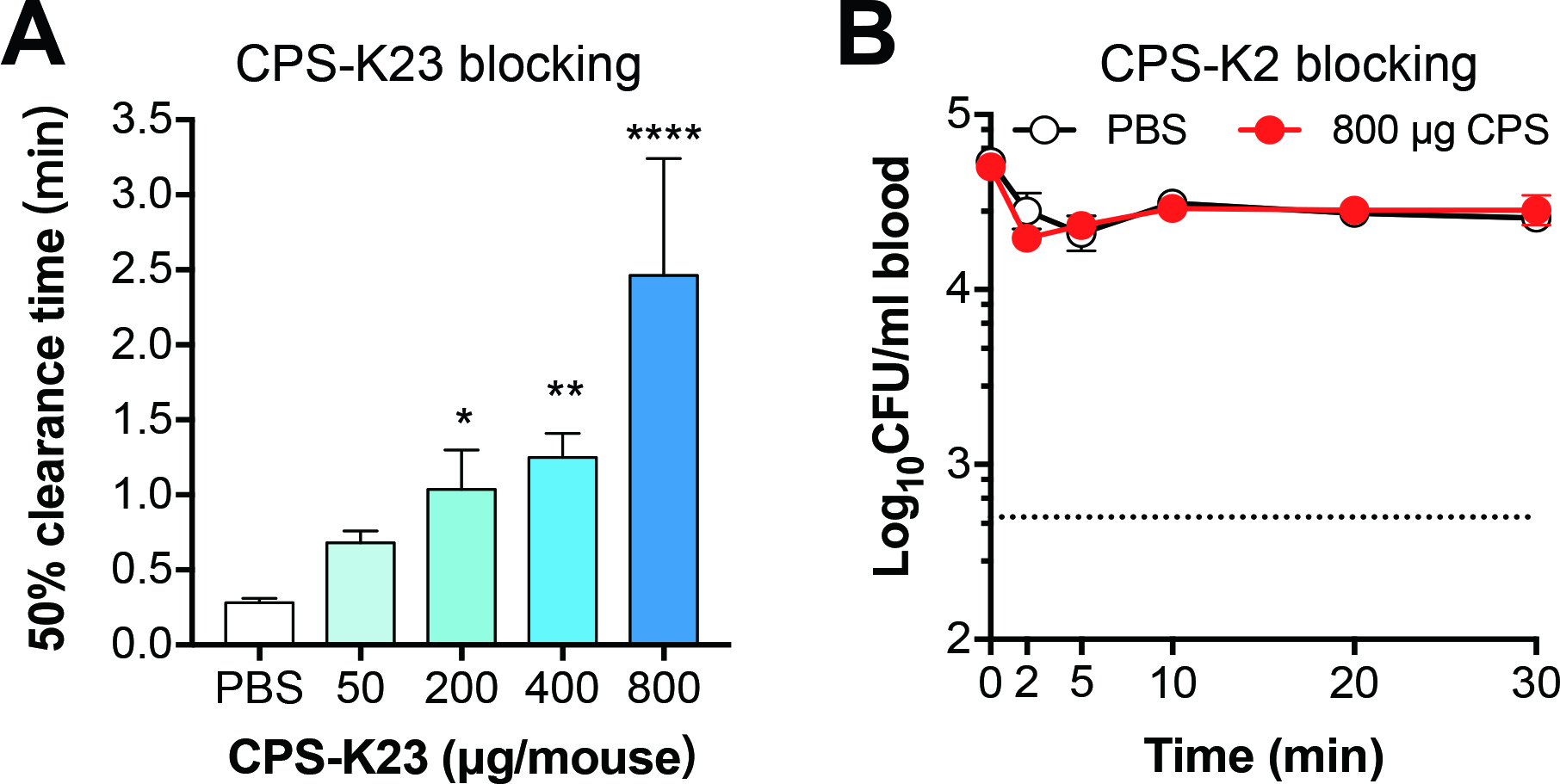

Supplement: S4 Fig — 50% clearance time of LV K. pneumoniae (K23) in mice with CPS pretreatment (related to Fig 5) (A). Time to clearance of 50% inoculum from blood was calculated based on nonlinear regression analysis of early bacteremia data. CD1, n = 3. Effect of free K2 capsule on clearance of HV K2 strain (B). Mice were intravenously inoculated with PBS or 800 μg purified CPS from the K2 strain 2 min before i.v. infection with 105 CFU of K2. The bacterial loads in blood within 30 min were presented. CD1, n = 5. The data are presented as mean ± SD. Ordinary one-way ANOVA with Tukey’s multiple comparisons test (A) and two-way ANOVA with Tukey’s multiple comparisons test were performed (B). *, P < 0.1; **, P <0.01, ****, P <0.0001. (TIF) [file ppat.1010693.s004.tif]
