# Supplementary material for: Capsule type defines the capability of Klebsiella pneumoniae in evading Kupffer cell capture in the liver
Source: PLoS Pathog. 2022 Aug 1;18(8):e1010693. doi: 10.1371/journal.ppat.1010693 (PMC9342791; doi:10.1371/journal.ppat.1010693)
Supplement: S3 Table — (DOCX) [file ppat.1010693.s007.docx]

**S3 Table. Laboratory strains and derivatives used in this study**

| **Strain** | **Description** | **Reference or source** |
| --- | --- | --- |
| DH5α | *Escherichia coli* DH5α | NEB |
| ATCC 43816 | *Klebsiella pneumoniae* standard strain, serotype K2 | ATCC |
| ATCC 13883 | *K. pneumoniae* standard strain, serotype K3 | ATCC |
| ATCC 35657 | *K. pneumoniae* standard strain, serotype KL64 | ATCC |
| ATCC BAA-1144 | *K. pneumoniae* standard strain, untypable by *wzi* sequencing | ATCC |
| NTUH-2044 | *K. pneumoniae* standard strain, serotype K1 | [1] |
| TH13179 | *K. pneumoniae* ATCC 43816 derivative; carrying pCasKP-apr; Apr^R^ | This study |
| TH13416 | *E. coli* DH5α derivative; carrying pSGKP-spe with *cps* spacer targeting *galF* of ATCC 43816 or TH12849; Spe^R^ | This study |
| TH13692 | ATCC 43816 derivative; ∆*galF*-*wzc*; carrying pCasKP-apr; Apr^R^ | This study |
| TH13609 | *E. coli* DH5α derivative; carrying pSGKP-spe with *cps* spacer targeting *wcuF* of ATCC 43816; Spe^R^ | This study |
| TH13811 | ATCC 43816 derivative; ∆*galF*-*wcaJ*; carrying pCasKP-apr; Apr^R^ | This study |
| TH13847 | *E. coli* DH5α derivative; carrying pSGKP-spe with *cps* spacer targeting *wcaJ* of ATCC 43816; Spe^R^ | This study |
| TH13863 | ATCC 43816 derivative; ∆*cps*K2; carrying pCasKP-apr; Apr^R^ | This study |
| *∆cps* | ATCC 43816 derivative; ∆*cps*K2 | This study |
| TH13818 | *E. coli* DH5α derivative; carrying pSGKP-spe with spacer targeting the junction of up- and downstream *cps* sequences in TH13863; Spe^R^ | This study |
| K2^K1^ | ATCC 43816 derivative; capsule switched strain; ∆*cps*K2::*cps*K1 | This study |
| K2^K2^ | ATCC 43816 derivative; capsule revertant; ∆*cps*K2::*cps*K2 | This study |
| K2^K3^ | ATCC 43816 derivative; capsule switched strain; ∆*cps*K2::*cps*K3 | This study |
| K2^K23^ | ATCC 43816 derivative; capsule switched strain; ∆*cps*K2::*cps*K23 | This study |
| TH15640 | TH12849 (K3) derivative; carrying pCasKP-apr; Apr^R^ | This study |
| TH16554 | TH12849 (K3) derivative; ∆*cps*K3; carrying pCasKP-apr; Apr^R^ | This study |
| TH16545 | *E. coli* DH5α derivative; carrying pSGKP-spe with spacer targeting the junction of up- and downstream *cps* sequences in TH16554; Spe^R^ | This study |
| TH16675 | TH12849 (K3) derivative; Transitional strain; *∆cps*K3::K2(1 kb)-*kan^R^*-K2(1 kb); carrying pCasKP-apr; Apr^R^ and Km^R^ | This study |
| TH16543 | *E. coli* DH5α derivative; carrying pSGKP-spe with spacer targeting kanamycin resistant gene (*kan^R^*); Spe^R^ | This study |

**S3 Table. Laboratory strains and derivatives used in this study (Continued)**

| **Strain** | **Description** | **Reference or source** |
| --- | --- | --- |
| K3^K2^ | TH12849 (K3) derivative; capsule switched strain; *∆cps*::*cps*K2 | This study |
| TH16672 | TH12880 (K7) derivative; carrying pCasKP-apr; Apr^R^ | This study |
| TH16724 | *E. coli* DH5α derivative; carrying pSGKP-spe with *cps* spacer targeting *galF* of TH12880 (K7); Spe^R^ | This study |
| TH16726 | TH12880 (K7) derivative; Transitional strain; *∆cps*K7::K2(1 kb)-K2(1 kb); carrying pCasKP-apr and pTH16724; Apr^R^ and Spe^R^ | This study |
| TH16729 | *E. coli* DH5α derivative; carrying pSGKP-spe with spacer targeting the junction of up- and downstream *cps* sequences in TH16726; Spe^R^ | This study |
| K7^K2^ | TH12880 (K7) derivative; capsule switched strain; ∆*cps*K7::*cps*K2 | This study |
| TH16549 | ATCC 43816 derivative; Transitional strain;*∆cps*K2::K47 (1 kb)-*kan^R^*-K47 (1 kb); carrying pCasKP-apr; Apr^R^ and Kan^R^ | This study |
| K2^K47-L^ | ATCC 43816 derivative; capsule switched strain; *∆cps*K2::*cps*K47-L | This study |
| K2^K47-H^ | ATCC 43816 derivative; capsule switched strain; *∆cps*K2::*cps*K47-H | This study |
| TH16712 | ATCC 43816 derivative; capsule switched strain; *∆cps*K2::*cps*K47-H; carrying pCasKP-apr; Apr^R^ | This study |
| TH16544 | *E. coli* DH5α derivative; carrying pSGKP-spe with spacer targeting inserted sequence (IS) in *cps* of K2^K47-H^; Spe^R^ | This study |
| K2^K47-H^*^∆^*^IS^ | ATCC 43816 derivative; *∆cps*K2::*cps*K47-H*∆*IS | This study |
| TH16547 | *E. coli* DH5α derivative; carrying pSGKP-spe with spacer targeting *wzc* in *cps* locus of K2^K47-H^; Spe^R^ | This study |
| TH16707 | ATCC 43816 derivative; *∆cps*K2::*cps*K47-H*∆wzc*; carrying pCasKp-apr; Apr^R^ | This study |
| TH16709 | *E. coli* DH5α derivative; carrying pSGKP-spe with spacer targeting the junction of up- and downstream sequences flanking *wzc* in TH16707; Spe^R^ | This study |
| K2^K47-H (C→G)^ | ATCC 43816 derivative; *∆cps*K2::*cps*K47-H (C→G) | This study |

Apr^R^: apramycin resistance; Spe^R^: spectinomycin resistance; *kan^R^*: kanamycin resistant gene; ATCC: American Type Culture Collection; NEB: New England Biolabs (Beijing) LTD

**SI references**

1. Chou HC, Lee CZ, Ma LC, Fang CT, Chang SC, Wang JT. Isolation of a chromosomal region of *Klebsiella pneumoniae* associated with allantoin metabolism and liver infection. Infect Immun. 2004;72(7):3783-92. Epub 2004/06/24. doi: 10.1128/IAI.72.7.3783-3792.2004. PubMed PMID: 15213119; PubMed Central PMCID: PMCPMC427404.
