# Supplementary material for: Capsule type defines the capability of Klebsiella pneumoniae in evading Kupffer cell capture in the liver
Source: PLoS Pathog. 2022 Aug 1;18(8):e1010693. doi: 10.1371/journal.ppat.1010693 (PMC9342791; doi:10.1371/journal.ppat.1010693)
Supplement: S4 Table — (DOCX) [file ppat.1010693.s008.docx]

**S4 Table. Primers used in this study**

| **Primer ID** | **Sequence (5’-3’)** | **Description** |
| --- | --- | --- |
| *wzi*_for2  (Pr14753) | GTGCCGCGAGCGCTTTCTATCTTGGTATTCC | Forward primer to amplify *wzi* gene for capsule typing |
| *wzi*_rev  (Pr14754) | GAGAGCCACTGGTTCCAGAA[C/T]TT[C/G]ACCGC | Reverse primer to amplify *wzi* gene for capsule typing |
| Pr15206 | tagtGGCCACAAAGGCAATTCCAA | Forward sequence to form spacer targeting *galF* in ATCC 43816 *cps* locus |
| Pr15207 | aaacTTGGAATTGCCTTTGTGGCC | Reverse sequence to form spacer targeting *galF* in ATCC 43816 *cps* locus |
| Pr15230 | TCAATCATGACCGAGAAGCCAATG | Forward primer to amplify upstream homologous arm of *cps* locus |
| Pr15231 | CTCCGTTATCCTGACAACTGCGATCCGCCCTATTCTG | Reverse primer to amplify upstream homologous arm of ATCC 43816 *cps* locus |
| Pr15232 | GGGCGGATCGCAGTTGTCAGGATAACGGAGGTTACATAGAAGGG | Forward primer to amplify downstream homologous arm of ATCC 43816 *cps* locus |
| Pr15233 | GAAGGGCATGATGTTGTCGGAATC | Reverse primer to amplify downstream homologous arm of *cps* locus |
| Pr15236 | CCCTTCTTGAGTAACAACTGCGATCCGCCCTATTCTG | Reverse primer to amplify upstream homologous arm of ATCC 43816 *cps* locus |
| Pr15237 | GGGCGGATCGCAGTTGTTACTCAAGAAGGGGACGAAGAATCC | Forward primer to amplify downstream homologous arm of the first *cps* region |
| Pr15238 | GATAGTATGTGAATCCATTATCTCAACAACTTTATTAG | Reverse primer to amplify downstream homologous arm of the first *cps* region |
| Pr15425 | tagtCATTTGCCGGTTTTATTGTA | Forward sequence to form spacer targeting *wcuF* in ATCC 43816 *cps* locus |
| Pr15426 | aaacTACAATAAAACCGGCAAATG | Reverse sequence to form spacer targeting *wcuF* in ATCC 43816 *cps* locus |
| Pr15578 | ATCCTGCTAATGCTCTAACTGCGATCCGCCCTATTCTG | Reverse primer to amplify upstream homologous arm of ATCC 43816 *cps* locus |
| Pr15579 | AGGGCGGATCGCAGTTAGAGCATTAGCAGGATTTTTTAGACGGAT | Forward primer to amplify downstream homologous arm of the second *cps* region |
| Pr15580 | GAGATCTAGACTTGTCTTAAGGAGCATCTAATCAATAAGCAG | Reverse primer to amplify downstream homologous arm of the second *cps* region |
| Pr15592 | tagtACCAGCAGGAATCAGTTTGC | Forward sequence to form spacer targeting *wcaJ* in ATCC 43816 *cps* locus |
| Pr15593 | aaacGCAAACTGATTCCTGCTGGT | Reverse sequence to form spacer targeting *wcaJ* in ATCC 43816 *cps* locus |
| Pr15554 | tagtGATCGCAGTTGTCAGGATAA | Forward sequence to form spacer targeting the junction sequence of TH13863 |
| Pr15555 | aaacTTATCCTGACAACTGCGATC | Reverse sequence to form spacer targeting the junction sequence of TH13863 |
| Pr15715 | AAAGGCAAAATGGGTGCGGG | Forward primer to amplify *cps* locus |
| Pr15716 | CCAAATAGAACGGCAGCGTATTATAACTC | Reverse primer to amplify *cps* locus from NTUH-2044 (K1) |
| Pr15764 | GCTCATCACGCCCTTACATGCAA | Reverse primer to amplify *cps* locus from ATCC13883 (K3) |
| Pr17015 | GGCAAAAACCGCATCGCTATTG | Forward primer to amplify *cps* locus from TH12852 (K23) |
| Pr17016 | CCATGACGATATCCTTAACCGCTGC | Reverse primer to amplify *cps* locus from TH12852 |

**S4 Table. Primers used in this study (Continued)**

| **Primer ID** | **Sequence (5’-3’)** | **Description** |
| --- | --- | --- |
| Pr15761 | ATGCAGTCACCATTATGGCGAGC | Reverse primer to amplify *cps* locus from K47 stains |
| Pr18948 | tagtATCTATAAAATATATTTTAT | Forward sequence to form spacer targeting *kan^R^* |
| Pr18949 | aaacATAAAATATATTTTATAGAT | Reverse sequence to form spacer targeting *kan^R^* |
| Pr19053 | tagtTTGCGCCATTTAAAAAACTC | Forward sequence to form spacer targeting IS sequence in TH12845 (K47-H) *cps* locus |
| Pr19054 | aaacGAGTTTTTTAAATGGCGCAA | Reverse sequence to form spacer targeting IS sequence in TH12845 *cps* locus |
| Pr19055 | tagtGGATCGCAGTTGATTGGAAA | Forward sequence to form spacer targeting *galF* in TH12849 (K3) *cps* locus |
| Pr19056 | aaacTTTCCAATCAACTGCGATCC | Reverse sequence to form spacer targeting *galF* in TH12849 *cps* locus |
| Pr19059 | tagtTGGTTACTGATATAATGACA | Forward sequence to form spacer targeting *wzc* in TH12845 *cps* locus |
| Pr19060 | aaacTGTCATTATATCAGTAACCA | Reverse sequence to form spacer targeting *wzc* in TH12845 *cps* locus |
| Pr19061 | AACTGCGATCCGCCCTATTCTG | Reverse primer to amplify the upstream homologous arm of *cps* locus |
| Pr19062 | AAGTGAGTGCAGAATAGGGCGGATC | Forward primer to amplify the upstream homologous arm of *cps* locus |
| Pr19063 | TTATCCTTCAAGGAGTTTTCAGCATTATCCGACGGACTGCTTCTTCGCCAGTT | Reverse primer to amplify the upstream homologous arm of *cps* locus |
| Pr19064 | GGATAATGCTGAAAACTCCTTGAAGGAT | Forward primer to amplify the *kan^R^* |
| Pr19065 | TTTTTATTTTTGGTGAATTCTAGGTACTAAAACAAT | Reverse primer to amplify the *kan^R^* |
| Pr19066 | TTAGTACCTAGAATTCACCAAAAATAAAAAGCATCAAGGCGAAAGGTATTCCG | Forward primer to amplify the downstream homologous arm of *cps* locus |
| Pr19067 | ACCCCTTCTATGTAACCTCCGTTATCCT | Reverse primer to amplify the downstream homologous arm of TH12845 *cps* locus |
| Pr19068 | GTCAGGATAACGGAGGTTACATAGAAGGG | Forward primer to amplify the downstream homologous arm of ATCC 43816 *cps* locus |
| Pr19075 | CATGCCGTTTCCAATCAACTGCGATCCGCCCTATTCTG | Reverse primer to amplify the upstream homologous arm of TH12849 *cps* locus |
| Pr19076 | AGGGCGGATCGCAGTTGATTGGAAACGGCATGGATACAGC | Forward primer to amplify the downstream homologous arm of TH12849 *cps* locus |
| Pr19080 | ATGCGAGCTGGTACAGACATTACGG | Forward primer to amplify IS sequence from TH12846 (K47-L) |
| Pr19081 | TTGAAGCGAGATCCGTTGCTGAT | Reverse primer to amplify IS sequence from TH12846 (K47-L) |
| Pr19084 | CATGCCGTTTCCAATCGCGAAAACAGCATTATATGCTGATAGATAAAA | Reverse primer to amplify the downstream homologous arm of ATCC 43816 *cps* locus |
| Pr19085 | ATAATGCTGTTTTCGCGATTGGAAACGGCATGGATACAGC | Forward primer to amplify the downstream homologous arm of TH12849 *cps* locus |
| Pr19091 | AAAGCCAATGTGACGCCCTATATGGATCTGGGATATCTCGCTG | Reverse primer to amplify the upstream homologous arm of TH12845 *wzc* |
| Pr19093 | CCAGATCCATATAGGGCGTCACATTGGCTTTATCAGATTTC | Reverse primer to amplify the downstream homologous arm of TH12845 *wzc* |

**S4 Table. Primers used in this study (Continued)**

| **Primer ID** | **Sequence (5’-3’)** | **Description** |
| --- | --- | --- |
| Pr19094 | CAGCGAGATATCCCAGATCCATATAGG | Forward primer to amplify TH12845 *wzc* |
| Pr19096 | CCAATATGAGCCGAACCAATTACAGTTAT | Forward primer to amplify the upstream homologous arm of TH12845 *wzc* |
| Pr19097 | CACATAATATTGTATTCGACGGGAAGCC | Reverse primer to amplify the downstream homologous arm of TH12845 *wzc* |
| Pr19100 | tagtTAGCATGGGCAGCGTCACAT | Forward sequence to form spacer targeting the junction sequence of TH16707 |
| Pr19101 | aaacATGTGACGCTGCCCATGCTA | Reverse sequence to form spacer targeting the junction sequence of TH16707 |
| Pr19122 | AGCGTCATATTGGCGGAGAG | Forward primer to amplify upstream homologous arm of TH12880 *cps* locus |
| Pr19123 | GAATTATGCCGGTTTTGTGAGCG | Reverse primer to amplify upstream homologous arm of TH12880 *cps* locus |
| Pr19124 | CGCTCACAAAACCGGCATAATTC | Forward primer to amplify upstream homologous arm of ATCC 43816 *cps* locus |
| Pr19125 | GCTTCGGTGGAGTCGGTAAAAGGACCACCACAAACGGGT | Reverse primer to amplify upstream homologous arm of ATCC 43816 *cps* locus |
| Pr19126 | TTTACCGACTCCACCGAAGC | Forward primer to amplify downstream homologous arm of *cps* locus |
| Pr19127 | TCATTCTGCGTTTTGGCATGG | Reverse primer to amplify downstream homologous arm of *cps* locus |
| Pr19128 | CCATGCCAAAACGCAGAATGA | Forward primer to amplify downstream homologous arm of TH12880 *cps* locus |
| Pr19129 | GTTCACGAAAGCGATGCTGG | Reverse primer to amplify downstream homologous arm of TH12880 *cps* locus |
| Pr19130 | tagtCCAGACCAAAGAGCCGATGG | Forward sequence to form spacer targeting *galF* in TH12880 cps locus |
| Pr19131 | aaacCCATCGGCTCTTTGGTCTGG | Reverse sequence to form spacer targeting *galF* in TH12880 cps locus |
| Pr19132 | tagtGGTAAAAGGACCACCACAAA | Forward sequence to form spacer targeting the junction sequence of TH16554 |
| Pr19133 | aaacTTTGTGGTGGTCCTTTTACC | Reverse sequence to form spacer targeting the junction sequence of TH16554 |
| *gapA*-F | GTTTTCCCAGTCACGACGTTGTATGAAATATGACTCCACTCACGG | Forward primer to amplify *gapA* for MLST of *K. pneumoniae* |
| *gapA*-R | TTGTGAGCGGATAACAATTTCCTTCAGAAGCGGCTTTGATGGCTT | Reverse primer to amplify *gapA* for MLST of *K. pneumoniae* |
| *infB*-F | GTTTTCCCAGTCACGACGTTGTACTCGCTGCTGGACTATATTCG | Forward primer to amplify *infB* for MLST of *K. pneumoniae* |
| *infB*-R | TTGTGAGCGGATAACAATTTCCGCTTTCAGCTCAAGAACTTC | Reverse primer to amplify *infB* for MLST of *K. pneumoniae* |
| *mdh*-F | GTTTTCCCAGTCACGACGTTGTACCCAACTCGCTTCAGGTTCAG | Forward primer to amplify *mdh* for MLST of *K. pneumoniae* |
| *mdh*-R | TTGTGAGCGGATAACAATTTCCCGTTTTTCCCCAGCAGCAG | Reverse primer to amplify *mdh* for MLST of *K. pneumoniae* |
| *pgi*-F | GTTTTCCCAGTCACGACGTTGTAGAGAAAAACCTGCCTGTACTGCTGGC | Forward primer to amplify *pgi* for MLST of *K. pneumoniae* |
| *pgi*-R | TTGTGAGCGGATAACAATTTCCGCGCCACGCTTTATAGCGGTTAAT | Reverse primer to amplify *pgi* for MLST of *K. pneumoniae* |
| *phoE*-F | GTTTTCCCAGTCACGACGTTGTAACCTACCGCAACACCGACTTCTTCGG | Forward primer to amplify *phoE* for MLST of *K. pneumoniae* |

**S4 Table. Primers used in this study (Continued)**

| **Primer ID** | **Sequence (5’-3’)** | **Description** |
| --- | --- | --- |
| *phoE*-R | TTGTGAGCGGATAACAATTTCTGATCAGAACTGGTAGGTGAT | Reverse primer to amplify *phoE* for MLST of *K. pneumoniae* |
| *Vic*-F | GTTTTCCCAGTCACGACGTTGTAGGCGAAATGGCWGAGAACCA | Forward primer to amplify *rpoB* for MLST of *K. pneumoniae* |
| *Vic*-R | GTTTTCCCAGTCACGACGTTGTATGAAATATGACTCCACTCACGG | Reverse primer to amplify *rpoB* for MLST of *K. pneumoniae* |
| *tonB*-F | GTTTTCCCAGTCACGACGTTGTACTTTATACCTCGGTACATCAGGTT | Forward primer to amplify *tonB* for MLST of *K. pneumoniae* |
| *tonB*-R | TTGTGAGCGGATAACAATTTCATTCGCCGGCTGRGCRGAGAG | Reverse primer to amplify *tonB* for MLST of *K. pneumoniae* |
| Mseq-F | GTTTTCCCAGTCACGACGTTGTA | Forward primer to sequence amplified genes for MLST of *K. pneumoniae* |
| Mseq-R | TTGTGAGCGGATAACAATTTC | Reverse primer to sequence amplified genes for MLST of *K. pneumoniae* |
