# Supplementary material for: Capsule type defines the capability of Klebsiella pneumoniae in evading Kupffer cell capture in the liver
Source: PLoS Pathog. 2022 Aug 1;18(8):e1010693. doi: 10.1371/journal.ppat.1010693 (PMC9342791; doi:10.1371/journal.ppat.1010693)
Supplement: S5 Table — (DOCX) [file ppat.1010693.s009.docx]

**S5 Table.** **Information for constructions of strains used in this study**

| **Strain ID** | | **Carrying plasmid** | | **Donor DNA used for construction of plasmid** | | | | | | | | | | | **Recipient strain** | | |
| --- | --- | --- | --- | --- | --- | --- | --- | --- | --- | --- | --- | --- | --- | --- | --- | --- | --- |
|  |  |  |  | **Plasmids** | | | | **Insertion spacer** | | **Digestion** | | | | |  |  |  |
| TH13416 | | pSGKP-spe with *cps* spacer targeting *galF* | | pSGKP-spe | | | Pr15206/15207 | | | | BsaI | | | DH5α | | |  |
| TH13609 | | pSGKP-spe with *cps* spacer targeting *wcuF* | | pSGKP-spe | | | Pr15425/15426 | | | | BsaI | | | DH5α | | |  |
| TH13847 | | pSGKP-spe with *cps* spacer targeting *wcaJ* | | pSGKP-spe | | | Pr15592/15593 | | | | BsaI | | | DH5α | | |  |
| TH13818 | | pSGKP-spe with spacer targeting the junction sequence in TH13863 | | pSGKP-spe | | | Pr15554/15555 | | | | BsaI | | | DH5α | | |  |
| TH16545 | | pSGKP-spe with *cps* spacer targeting *galF* of TH12849 | | pSGKP-spe | | | Pr19055/19056 | | | | BsaI | | | DH5α | | |  |
| TH16543 | | pSGKP-spe with spacer targeting *kan^R^* | | pSGKP-spe | | | Pr18948/18949 | | | | BsaI | | | DH5α | | |  |
| TH16544 | | pSGKP-spe with spacer targeting IS of TH12845 | | pSGKP-spe | | | Pr19053/19054 | | | | BsaI | | | DH5α | | |  |
| TH16547 | | pSGKP-spe with spacer targeting *wzc* of TH12845 | | pSGKP-spe | | | Pr19059/19060 | | | | BsaI | | | DH5α | | |  |
| TH16709 | | pSGKP-spe with spacer targeting the junction sequence of TH16707 | | pSGKP-spe | | | Pr19100/19101 | | | | BsaI | | | DH5α | | |  |
| TH16724 | | pSGKP-spe with *cps* spacer targeting *galF* of TH12880 | | pSGKP-spe | | | Pr19130/19131 | | | | BsaI | | | DH5α | | |  |
| TH16729 | | pSGKP-spe with spacer targeting the junction sequence of TH16726 | | pSGKP-spe | | | Pr19132/19133 | | | | BsaI | | | DH5α | | |  |
| **Strain ID** | | **Genotype** | **Donor DNA used for mutagenesis** | | | | | | **Template**  **DNA** | | | **Recipient or parental strain** | | | | **Curing plasmid** | |
|  |  |  | **Plasmid** | | | **Repair template** | | |  |  |  |  |  |  |  |  |  |
| TH13179 | | ATCC43816:pCasKP-apr | pCasKP-apr | | | / | | | / | | | ATCC 43816 | | | | / | |
| TH13692 | | ATCC43816 ∆*galF-wzc*; pCasKP-apr | pTH13416 | | | Pr15230/15236^a^  Pr15237/15238^b^ | | | ATCC 43816 | | | TH13179 | | | | pTH13416 | |
| TH13811 | | ATCC43816 ∆*galF*-*wcaJ*; pCasKP-apr | pTH13609 | | | Pr15230/15578^a^  Pr15579/15580^b^ | | | ATCC 43816 | | | TH13692 | | | | pTH13609 | |
| TH13863 | | ATCC43816 ∆*galF*-*ugd*; pCasKP-apr | pTH13847 | | | Pr15230/15231^a^  Pr15232/15233^b^ | | | ATCC 43816 | | | TH13811 | | | | pTH13847 | |
| *∆cps* | | ATCC43816 ∆*galF*-*ugd* (whole *cps* locus) | / | | | / | | | / | | | TH13863 | | | | pCasKP-apr | |
| K2^K3^ | | ATCC43816 ∆*cps*K2::*cps*K3 | pTH13818 | | | Pr15715/15764^c^ | | | ATCC 13883 | | | TH13863 | | | | pCasKP-apr; pTH13818 | |
| K2^K1^ | | ATCC43816  ∆*cps* K2::*cps*K1 | pTH13818 | | | Pr15715/15716 ^c^ | | | NTUH-2044 | | | TH13863 | | | | pCasKP-apr; pTH13818 | |
| K2^K23^ | ATCC43816 ∆*cps*::*cps*K23 | | pTH13818 | | Pr17015/17016 ^c^ | | | | TH12852 | | | | TH13863 | | | pCasKP-apr; pTH13818 | |
| K2^K2^ | ATCC43816 ∆*cps*::*cps*K2 | | pTH13818 | | Pr15230/15233^c^ | | | | ATCC 43816 | | | | TH13863 | | | pCasKP-apr; pTH13818 | |
| TH15640 | TH12849(K3):pCasKP-apr | | pCasKP-apr | | / | | | | / | | | | TH12849 | | | / | |

**S5 Table. Information for constructions of strains used in this study (Continued)**

| **Strain ID** | **Genotype** | | **Donor DNA used for mutagenesis** | | **Template**  **DNA** | **Recipient or parental strain** | **Curing plasmid** |
| --- | --- | --- | --- | --- | --- | --- | --- |
|  |  |  | **Plasmid** | **Repair template** |  |  |  |
| TH16554 | TH12849 ∆*cps*; pCasKP-apr | | pTH13416 | Pr15230/19075^a^  Pr19076/15233^b^ | TH12849 | TH15640 | pTH13416 |
| TH16675 | TH12849 ∆*cps*::  K2 (1 kb) -*kan^R^*-K2 (1 kb);  pCasKP-apr | | pTH16545 | Pr15230/19061^a^  Pr19062/19063^d^  Pr19064/19065^f^  Pr19066/19084^e^  Pr19085/15233^b^ | TH12849;  ATCC 43816 | TH16554 | pTH16545 |
| K3^K2^ | TH12849 *∆cps*K3::*cps*K2 | | pTH16543 | Pr15230/15233^c^ | ATCC 43816 | TH16675 | pCasKP-apr; pTH16543 |
| TH16672 | TH12880(K7):pCasKP-apr | | pCasKP-apr | / | / | TH12880 | / |
| TH16726 | TH12880 *∆cps*K7::  K2 (1 kb)-K2 (1 kb);  pCasKP-apr | | pTH16724 | Pr19122/19123^a^  Pr19124/19125^d^  Pr19126/19127^e^  Pr19128/19129^b^ | TH12880;  ATCC 43816 | TH16672 | pTH16543 |
| K7^K2^ | TH12880 *∆cps*::*cps*K2 | | pTH16729 | Pr19124/19127 | ATCC 43816 | TH16726 | pCasKP-apr;  pTH16729 |
| TH16549 | ATCC43816 *∆cps*K2::  K47 (1 kb)-*kan^R^*-K47 (1 kb); pCasKP-apr | | pTH13818 | Pr15230/19061^a^  Pr19062/19063^d^  Pr19064/19065^f^  Pr19066/19067^e^  Pr19068/15233^b^ | ATCC 43816; TH12845 | TH13863 | pTH13818 |
| K2^K47-L^ | ATCC43816  *∆cps*K2::*cps*K47-L | | pTH16543 | Pr15715/15761^c^ | TH12846 | TH16549 | pCasKP-apr; pTH16543 |
| K2^K47-H^ | ATCC43816  *∆cps*K2::*cps*K47-H | | pTH16543 | Pr15715/15761^c^ | TH12845 | TH16549 | pCasKP-apr; pTH16543 |
| TH16712 | ATCC43816  *∆cps*K2::*cps*K47-H;  pCasKP-apr | | pCasKP-apr | / | / | TH16711 | / |
| K2^K47-H∆IS^ | | ATCC43816 *∆cps*K2::*cps*K47-H*∆*IS | pTH16544 | Pr19080/19081 | TH12846 | TH16712 | pCasKP-apr; pTH16544 |
| TH16707 | | ATCC43816 *∆cps*K2::*cps*K47-H*∆wzc*; pCasKP-apr | pTH16547 | Pr19096/19091^a^  Pr19093/19097^b^ | TH12845 | TH16712 | pTH16547 |
| K2 ^K47-H (C→G)^ | | ATCC43816 *∆cps*K2:: *cps*K47-H (C→G) | pTH16709 | Pr19094/19097 | TH12846 | TH16707 | pCasKP-apr;  pTH16709 |

^a^ Primers used to amplify upstream homologous arm from recipient strain

^b^ Primers used to amplify downstream homologous arm from recipient strain

^c^ Primers used to amplify donor *cps* locus

^d^ Primers used to amplify upstream homologous arm from donor strain

^e^ Primers used to amplify downstream homologous arm from donor strain

^f^ Primers used to amplify Kanamycin resistant gene
