# Supplementary material for: Capsule type defines the capability of Klebsiella pneumoniae in evading Kupffer cell capture in the liver
Source: PLoS Pathog. 2022 Aug 1;18(8):e1010693. doi: 10.1371/journal.ppat.1010693 (PMC9342791; doi:10.1371/journal.ppat.1010693)
Supplement: S6 Table — (DOCX) [file ppat.1010693.s010.docx]

**S6 Table. Plasmids used in this study**

| **Plasmid** | **Description^a^** | **Reference or source** |
| --- | --- | --- |
| pCasKP-apr | Thermosensitive plasmid carrying Cas9 gene and lambda red system; Apr^R^ | [1] |
| pSGKP-spe | Expressing sgRNA and carrying *sacB*; Spe^R^ | [1] |
| pTH13416 | pSGKP-spe derivative with spacer Pr15206/15207 targeting *galF*; Spe^R^ | This study |
| pTH13609 | pSGKP-spe derivative with spacer Pr15425/15426 targeting *wcuF*; Spe^R^ | This study |
| pTH13847 | pSGKP-spe derivative with spacer Pr15592/15593 targeting *wcaJ*; Spe^R^ | This study |
| pTH13818 | pSGKP-spe derivative with spacer Pr15552/15553 targeting the junction sequence in TH13863; Spe^R^ | This study |
| pTH16545 | pSGKP-spe derivative with spacer Pr19055/19056 targeting *galF* in K3 strains; Spe^R^ | This study |
| pTH16543 | pSGKP-spe derivative with spacer Pr18948/18949 targeting *kan^R^*; Spe^R^ | This study |
| pTH16544 | pSGKP-spe derivative with spacer Pr19053/19054 targeting IS in TH12845 (K47-H); Spe^R^ | This study |
| pTH16547 | pSGKP-spe derivative with spacer Pr19059/19060 targeting *wzc* in K47 strains; Spe^R^ | This study |
| pTH16709 | pSGKP-spe derivative with spacer Pr19100/19101 targeting the junction sequence in TH16707; Spe^R^ | This study |
| pTH16724 | pSGKP-spe derivative with spacer Pr19130/19131 targeting *galF* in K7 strains; Spe^R^ | This study |
| pTH16731 | pSGKP-spe derivative with spacer Pr19132/19133 targeting the junction sequence in TH16726; Spe^R^ | This study |

Apr^R^: carrying apramycin resistance gene; *sacB*: sucrose-sensitive gene (encoding *Bacillus subtilis* levansucrase); Spe^R^: carrying spectinomycin resistance gene

**SI references**

1. Wang Y, Wang S, Chen W, Song L, Zhang Y, Shen Z, et al. CRISPR-Cas9 and CRISPR-assisted cytidine deaminase enable precise and efficient genome editing in *Klebsiella pneumoniae*. *Appl Environ Microbiol*. 2018;**84**(23):e01834-18. Epub 2018/09/16. doi: 10.1128/AEM.01834-18. PubMed PMID: 30217854; PubMed Central PMCID: PMCPMC6238054.
