# Supplementary material for: Capsule type defines the capability of Klebsiella pneumoniae in evading Kupffer cell capture in the liver
Source: PLoS Pathog. 2022 Aug 1;18(8):e1010693. doi: 10.1371/journal.ppat.1010693 (PMC9342791; doi:10.1371/journal.ppat.1010693)
Supplement: Attachment — Submitted filename: Responses to Reviewer 2022-6-10.docx [file ppat.1010693.s015.docx]

**Responses to Reviewer’s Comments**

**Manuscript No:** PPATHOGENS-D-21-02447

### Title: Capsule Type Defines the Capability of *Klebsiella pneumoniae* in Evading Kupffer Cell Capture in the Liver

**Date:** June 9, 2022

We greatly appreciate the reviewer’s insightful evaluation of our manuscripts, and have revised the entire paper according to the comments by performing additional experiments and modifying the text/figures. With these valuable suggestions, the revised manuscript has been significantly improved. Two versions of the revised manuscript have been submitted as requested, and the followings are my responses to the review comments.

**Part I – Summary**

**Reviewer #1:**

**1. A major weakness of this manuscript is the near complete omission of the contribution of mucoviscosity to hypervirulent Kp. The parental strain (43816) for the hybrid K type strains is known to be** **hypermucoviscous (HMV). Because it is not known if HMV is dependent on capsule type, it is essential that capsule levels and mucoviscosity be quantified in the strains used. If the hybrid strains produce little capsule (less than donor strain) or are HMV negative, then the interpretation of capsule-type dependency is impossible to separate from capsule amount or HMV** **status.**

We agree with the reviewer on the importance of capsule quantity and mucoviscosity in *Klebsiella pneumoniae* pathogenesis, and have performed additional experiments to characterize these two traits in capsule type switched hybrid strains in this revision. The data show that neither capsule amount nor mucoviscosity is involved in the capsule type-dependent switch of the hybrid strains in virulence phenotypes (**Fig. 2C**, **2D**). The details of the data are described in our responses to related questions in the following sections. (page 7, lines 153-166).

**2.** **One potential strain to consider adding to the analysis is a hybrid strain with the K47 cps locus. As at least some of the K47 isolates were HV, this demonstrates that this capsule type has the potential for hypervirulence. The data presented within is interesting independent of the capsule levels/HMV status, but this information is critical for the current interpretation of capsule-type dependence for Kupffer cell clearance.**

We’ve selected two K47 strains with opposite virulence phenotypes, and transferred their capsular polysaccharide (*cps*) biosynthesis gene clusters to the *cps* locus of the K2 strain. The resulting K2 hybrid strains recapitulated the low- and high-virulence phenotypes of the donor K47 strains in bacterial clearance from the blood and capture in the liver. Further mutagenesis work revealed that the two sequence differences between the *cps* clusters are responsible for the phenotypic difference. It appears that the sequence differences make the low-virulence strain produce a dysfunctional capsule by impairing the elongation of the K47 capsular polysaccharide. This finding indicates that the K47 capsule is poorly recognized by Kupffer cells in the liver and thereby defines a high-virulence phenotype. We have incorporated these results in the new Figure 7 and revised text (pages 15-16, lines 395-440).

**Reviewer #2:**

**1.** **There are a few areas for improvement, namely improved validation the capsule-switched strains as well as enhancing the text by discussing potential role of complement and relevant past studies.**

We have addressed these issues by further characterization of the capsule-switched strains. The results show that the low- and high-virulence phenotypes of the capsule serotype derivatives are not caused by capsule amount and mucoviscosity (**Fig. 2C** and **D**; page 7, lines 153-166). We’ve found that the complement system is necessary for hepatic capture of acapsular mutant bacteria, but not low-virulence K23 bacteria (**Fig. 6E** and **G**; pages 14, lines 375-382).

**Reviewer #3:**

None

**Part II – Major Issues**

**Reviewer #1:**

**1. In figures reporting the % of the bacteria per organ, the CFU need to be reported as well as percent. The overall differences in CFU are interesting and important in addition to the percent per organ.**

The revision has switched to show the CFU values per organ, with the corresponding percent numbers in supplemental materials. The specific CFU values are listed in S2 Table.

**2. Do the hybrid K3 and K23 strains have similar or different bacterial burdens compared to K1 and K2?**

After replotting the data in Fig 3 and S2 Fig. in the revision, it is more readily to appreciate the bacterial levels of the hybrid strains in various organs and time points. The total bacterial burdens of the low-virulence (K2^K3^ and K2^K23^) and high-virulence (K2^K1^ and K2^K2^) strains were similar at 5 min post infection (revised **S2C Fig**), but the low-virulence strains were less abundant than the high-virulence strains at 30 min (revised **Fig 3D**). The result indicates significant killing of the LV hybrid strains after being capture in the liver. We have added this information in the text (page 9, lines 223-227).

**3. Did splenectomy impact overall burden numbers?**

The splenectomy did not appreciably affect the overall burden numbers as shown in Figure 4 and S2 Table.

**4. There is no mention in the Materials and Methods sections about replicate experiments. I noted that for many animal infections, the data are from a sample number of only 3. If this is indeed the complete data set, then the conclusions need to be softened. If these are representative experiments, then the M&M section must include information about reproducibility of the data.**

Revised in the figure legend.

**5. For the data generated with mice treated to reduce cell types, FACS analysis should be provided to indicate that the targeted cell types were indeed depleted. This is a necessary control to verify that the observed phenotypes are indeed due to depletion and not some other cause.**

The flow cytometry data are added in S4 Figure.

**6. A better definition of “capture” is desirable. Do you think KCs are rapidly phagocytosing all the bacteria or is it rapid binding followed by phagocytosis? Are there tissue culture assays that might help get a mechanistic idea of what this “capture” is?**

We have more clearly defined the word “capture” in the text (page 13, line 348); it is defined as KC’s recognition and binding of bacteria before phagocytosis and intracellular killing. We performed a new experiment to delineate the process of bacterial binding and phagocytosis (uptake) by KCs. Because KCs are embedded in the wall of the liver sinusoidal blood vessels and typically “damaged” after physical isolation from liver tissue, we used a published approach to assess bacterial binding and uptake of KCs by *in vivo* real-time imaging of pH-sensitive dye pHrodo-labeled bacteria in the liver sinusoids. The data show that KCs take up twice amount of acapsular bacteria (**Fig 5H**, Δ*cps*, 27.4%) as compared with the low-virulence counterpart (K2^K23^, 11.5%) in the first 30 min post intravenous infection. This finding is consistent with faster disappearance of Δ*cps* from the body (**Fig 3B**).  This information is described in the revised text (page 13, lines 338-348).

**7. How is ∆cps captured in the context of Fig. 5?**

We have experimentally addressed this question by two approaches. First, we tested if KCs differentially recognize the ∆*cps* and LV encapsulated strains by blocking hepatic capture of ∆*cps* with free capsular polysaccharide (CPS) of K23 or K108. Neither CPS showed significant effect. Second, we tested the contribution of scavenger receptor(s) and complement receptor CRIg to hepatic capture of ∆*cps,* which are known to recognize *Listeria monocytogenes* and/or *Staphylococcus aureus* in the liver. Hepatic capture of ∆*cps* was significantly impaired in mice pretreated with polyinosinic acid (poly(I)), a pan scavenger receptor inhibitor, and CRIg-deficient mice. We’ve concluded that the ∆*cps* strain is captured by KCs in a similar manner as other acapsular bacteria through recognizing the surface-exposed bacterial molecules/structures via KC’s pattern-recognition receptor (PRRs). These data are added in Fig 6 and revised text (page 14, lines 375-393).

**8.** **if purified CPS only blocks capture of cognate Kp, does this mean there are receptors for lots of different capsule types?**

Specific blocking of hepatic capture by free CPS indicates that KCs possess multiple capsule receptors. This conclusion is also supported by the data obtained in this revision in Fig 6, which K23 capsule is likely recognized by a scavenger receptor(s). It is possible that a single pattern recognition receptor recognizes the same motif of different capsular variants. As an example, human plasma L-ficolin recognizes acetylate monosaccharides on the capsules of multiple bacteria. We have added this point in the Discussion (pages 20-21, lines 529-559)

**9. Does the lack of capture of HV Kp suggest that the K1/K2 CPS does not interact with KCs? The interpretation of the data in Fig 6F would seem to suggest KCs are capable of recognizing K1/K2, at least after prior exposure (see comment below regarding this experiment).**

The poor hepatic capture of K1/K2 capsules strongly suggests that KCs are unable to recognize these capsules. Our new data provide supporting evidence that the poor hepatic capture of K2 capsule is not due to potential overload of the hepatic system with too many bacterial cells in the blood (**S4B Fig**.). Mice i.v. infected with lower infection doses of K2 bacteria (10^5^ CFU) showed a similar pattern of poor bacterial clearance as those infected with 5 × 10^6^ CFU (**Fig 3A**).

Our vaccination data strongly suggest that the whole cell immunization enables KCs to recognize the HV bacteria by eliciting pathogen-specific antibodies. This hypothesis is supported by effective capture of K2 bacteria pretreated with the immune serum from vaccinated mice (**Fig 8G**). This information is added in the revised text (pages 17-18, lines 483-488).

**10. The interpretation of the imaging in Fig 6F and movie S4 that KCs are responsible for capturing HV Kp is not convincing. There are numerous green dots not associated with the red staining, hinting that something else is capturing these bacteria. Staining for other cell types, or perhaps the use of the Clec4f-DTR mice could provide more convincing evidence.**

We have tested the possibility that other phagocytes other than KCs also capture HV bacteria in vaccinated mice by staining neutrophils in the IVM imaging, a phagocyte that is reported to kill KC-captured bacteria (Deniset et al, 2017). Quantification of the IVM data shows that 90% immobilized K2 bacteria in the liver sinusoids of vaccinated mice were associated with KCs, demonstrating that KCs are the major immune cells capturing the HV bacteria after immunization. Neutrophils occasionally passed the vasculatures but did not show detectable association with circulating or immobilized bacteria. K2 bacteria were occasionally deposited on the vascular wall of the liver sinusoids without apparent association with KCs. This might be caused by spatial out-of-focusing effect of bacterium-associated KCs or minor contribution of vascular endothelial cells to the vaccine-activated pathogen capture. We have accordingly revised Figure 8, Movie S4 and text (page 17, lines 471-483).

**Reviewer #2:**

**1. While the study uses capsule-switched recombinant strains, a strength, the only reported verification of these strains was through sequencing the recombined cps sequences. The study would benefit from additional verification of the production of capsule from these engineered heterologous strains, especially since they are largely associated with virulence-defective phenotypes.**

We’ve quantified the capsule production of the capsule-switched strains. The data revealed a comparable level of capsular polysaccharides in the isogenic K2 strains, supporting our conclusion that the capsule types are responsible for the low- and high-virulence phenotypes of *K. pneumoniae* strains (**Fig 2C** and **D**). The result is presented in Figure 2 with appropriate revision of the text (page 7, lines 153-166).

**Reviewer #3:**

**1. The aut****hors do not validate that ATCC 43816 K3 and K23 strains produce capsule. If the isogenic mutants do not produce comparable levels of capsule to K2 or K1, that could explain the observed phenotypes.**

Addressed as described in the above response to Reviewer #1.

**2. Most animal studies are done with 3 mice, some only have 2, some have more. The small sample size must be justified for providing adequate statistical power. This could be achieved using power calculations.**

Revised by repeating relevant experiments with additional mice and marking animal numbers in figure legends when necessary.

**3. Statistical analyses are poorly described and often not reported in the figure legends. It is not reported what statistical result is represented by the ****. It is hard to distinguish which results are non-significant vs not tested. And a lot of conclusions are made based on changes not reported to be statistically significant.**

Revised.

**4. All data are plotted as mean ± SEM according to the methods, but that is not reported in the figure legends. SEM does not provide information about the scatter of the data, only where the true mean lies and this seems a bit misleading considering most data points have only been performed in triplicate, once.**

Mean ± standard deviation (SD) is used in the revision.

**5. There are major issues with the transparency of how the data was calculated, challenging the interpretation of the data. For example, it is unclear how the denominator is calculated when % total bacteria is presented. How are total bacterial CFUs in a mouse determined when considering both solid organs and blood? At minimum, absolute CFU counts should be included in the supplement. However, it may help with clarity if instead absolute CFU counts for each organ/blood be presented in the figures and the changes be reported as fold change. Evaluating percentages for CFU data is challenges as a 99% decrease is only a 2-log decrease, so a lot of resolution is lost when using percentages.**

We apologize for the inadequate description of the calculation methodology. More detailed descriptions are added in figure legends and/or Materials/Methods. CFU counts are used in the figures to replace or supplement % presentation when necessary in revised figures. CFU values for individual organ/blood are presented in S2 Figure and S2 Table.

**6. Some specific challenges related to this data presentation as it stands:**

**1) It is hard to tell if the total liver counts are the same across bacterial species and altered blood counts are changing the liver percentages or are there actually less bacteria in the livers during HV infection.**

We have addressed this issue by presenting CFU counts of the liver and other each organ (instead of % total).

**2) Absolute counts would also be important in the splenectomy data as one of the organs contributing to the denominator is missing in half the groups.**

CFU counts are presented in the revised Figure 4.

**3)** **% inoculum in 3H makes 1-log increase in bacteria look huge compared to a 1-log decrease.**

% inoculum was changed to total CFU counts in the revised Figure 3E.

**7. Other issues with transparency in describing how the data were analyzed are below:**

**1) Time 0 CFU/ml in blood – is this the inoculum CFU/ml or the actual CFU/ml in the blood? It’s surprising that the time 0 CFU/ml in blood would be the same CFU/ml as the inoculum. I would expect it to be lower as the inoculum is diluted in the blood.**

Time 0 CFU/ml represents the actual CFU/ml blood. This value was obtained by dividing CFU with total blood volume (ml), which was estimated by the body weight of each mouse (1 gram body weight is equivalent to 0.07 ml blood; a 6-8 week-old mouse has a weight of 22-27 grams or blood volume of 1.5-1.9 ml). This information is added to the Materials and Methods.

**2) Fig 3I and 3K – the % inoculum in K doesn’t match the absolute counts in I. I would expect the ∆cps % inoculum to be 10^5 CFU/ml / 10^8 CFU/ml x100% = 0.1%**

We apologize for this confusion. The % inoculum was calculated by comparing the total CFU detected in the blood and five organs with the inoculum. This is clarified by labeling this and other equivalent figures with “% inoculum of total CFU”.

**3) When discussing changes “by XX%” it hard to know how that number was calculated. It might be clearer if these are reported as fold-change relative to the control condition.**

Fold charge is used for this type of description in the revision.

**4) Lines 350-355 and throughout the results section: The conclusion that Kupffer cells capture LV capsule types based on the CPS structure, isn’t fully supported by the data presented. How can this statement be supported by the evidence that often the ∆cps strain phenocopies LV strains without having a CPS?**

The LV capsule types mentioned here were encapsulated bacteria and does not include ∆*cps* strain. How ∆*cps* strain is caught is discussed as the response to question 7 of Reviewer #1 in Part II and this section was added in the revised text (page 14, lines 375-393).

.

**Part III – Minor Issues**

**Reviewer #1:**

**1. In general, I would recommend slightly larger figures, or at least larger text. Several were difficult to read due to the small text.**

Revised.

**2. Reference given for ATCC 43816 (line 122 Ref #36) does not seem correct?**

We have cited Fodah et al. (2014) for the strain although the paper we originally cited is listed as one of the source publications at the ATCC website.

**3. For some panels with infection data, the number of mice used is indicated, but for others this information is lacking. Please include for all. Figs. 3I-K indicates only 2 mice used? Cannot accurately do statistics with only 2 mice, so please remove asterisks if this number is correct.**

We’ve repeated the experiments with insufficient numbers of mice and added the number of mice in all the figures or legends.

**4. It is curious that the HV strains had a larger proportion of bacteria in the spleen than in the liver. As K1 strains are known to cause liver abscesses, some speculation on this result in the Discussion would be worth noting.**

This issue is elaborated in the revised Discussion (page 22, lines 587-596).

**5. Lines 194-195, an important clarification here is that LV bacteria have an advantage over acapsular bacteria.**

Revised (page 10, lines 246-248).

**6. Line 215, please expand the description of the Clec4f-DTR mice for readers not intimately familiar with this mutation/DTR treatment.**

Revised (page 12, lines 321-323).

**7. Lines 230-231, the statement that the ability of KCs to capture acapsular and LV Kp but not HV Kp explains the difference in these pathotypes is overstated. This is unlikely to be the only reason for the differences. Please make the language more suggestive than definitive.**

Revised (page 13, lines 348-351).

**8. It is assumed from Fig. 3 and beyond, that mostly the hybrid strains are used. It might be helpful to give these strains names to indicate that. The data in Fig. 5 uses LV isolates, but there is no change in nomenclature in the Results to distinguish this.**

The revised manuscript indicates the recipient strains with their capsular serotypes (e.g. K2), and the identities of the donor *cps* clusters with superscripted characters of the capsular serotypes of donor strains (e.g. K2^K1^).

**9. Line 366, referring to comment above about Fig 6F, this language should be altered. Also, “glued” is not the best term for bacteria interacting with host cells.**

Revised to “adhere” (page 23, line 625).

**10. Line 415,** **please state the route of infection for this LD50**

Revised (page 25, lines 668-669).

**11. In the** **Mouse infection section of the Materials & Methods, please provide some brief detail about the** **inoculations and surgeries. Please do not require your readers to find other papers to find the basic info for these procedures!**

Revised (pages 28, line 776-780).

**12. In the paragraph about preparation of inactivated bacteria for immunization, it would be helpful to include culture volume and final resuspension volumes, as well as inoculation volume for the killed bacteria.**

Revised (page 29, lines 799-804).

**13. Grammar & typos**

**Throughout, in describing fold change, “fold” is not plural**

**Line 142, perhaps “recovered” in place of “regrew”**

**Line 202, We, not W**

**Line 342, I’m not sure use of the word “act” is appropriate for the role of capsules. They provide protection, but not “act” on circulating phagocytes.**

**Line 363, inactivated**

**Line 378, “trial and error”**

**Line 482, ATCC 43816**

Revised.

**Reviewer #2:**

**1. Regarding the conclusions with the data using Clec4f-DTR mice, the authors state that the difference in hepatic capture "explains the drastic difference in virulence traits between these two types of the pathogen."**

**In addition to causing enhanced bacteremia levels and less early hepatic capture, does selective KC depletion with diphtheria toxin in transgenic mice allow LV strains to show the expected enhanced virulence (eg lower survival rate of infected mice) after the infection progresses past the early 30 minutes window studied?**

We performed infection experiment in Clec4f-DTR mice with 2 × 10^7^ CFU of serotype K23 strain. The KC-deficient mice became highly susceptible to the infection. The data are presented in Figure 5E (page 12, lines 327).

**2. If recombinant K1 or K2 HV capsule can be switched into a LV strain, can this mediate reversal of KC clearance and HV?**

We performed capsule switching in two LV strains (K3 and K7) with the HV capsule gene cluster of K2. Both the hybrid strains (K3^K2^ and K7^K2^) gained the capability of better survival in the bloodstream (**Fig 3I**) and escaping the hepatic capture (**Fig 3J**). This result has been added to the revised text (pages 10-11, lines 252-281).

**3. The possibility that the complement system, and different degrees of opsonization by complement with different capsule types, is a driver of different liver capture phenotypes should be discussed (in addition to the other mechanisms proposed by the authors in lines 350-357). Complement binding to diverse K. pneumoniae capsule types has been documented (eg see** **Jensen et al 2020), and it is possible that certain capsule structures interact with complement to create efficient KC opsonins, while interaction with other capsule structures does not efficiently opsonize.**

**Regarding the potential for complement interactions as a determinant of capture of invading bacteria by the liver and spleen, the authors should consider citing Spiegel****berg et al** **1963 and Brown & Frank 1981 (****Brown & Frank 1981 was cited in the manuscript but for a different purpose). These two papers also examined the role of liver vs spleen in rapid vs slow clearance of invasive organisms, another context in which these papers can be cited.**

We have performed new experiments to assess the role of the complement system in hepatic capture of low-virulence *K. pneumoniae.* C3-deficient mice did not show significant impairment in hepatic capture of K23 capsule type, a low-virulence type we chose for our focused investigation (Fig. 6E). It is still possible that the complement system is involved in hepatic capture of other capsule types. We have also elaborated this potential role of the complement system and cited the three papers in revised Discussion (page 20, lines 536-541).

**4. Figs 3-4: While showing the different tissue burdens as percentages (eg. Fig 3 panels D, E, G) is clear and useful, the interpretation of these data could benefit by providing total absolute counts of bacteria with each strain in the liver. This was done in Fig. S2A for the 5 min time point and was very useful. A similar supplemental graph with absolute liver counts at 30 minutes would be helpful to aid in understanding if there are still high absolute bacterial counts in the liver (as would be inferred since there are such high bacterial loads in bloodstream with HV strains at 30 min and beyond -- Fig. 3B, C).**

CFU counts are shown in the revised manuscript.

**2.** **The implication of this manuscript's findings for the propensity of certain HV K. pneumoniae infections to lead to pyogenic liver abscess could be discussed.**

Discussion is revised (page 22, lines 587-596).

**3. Line 311 states "****lethality of mice when** **high infection doses of LV Kp are used...", but mouse lethality/survival data were not shown in that context. This should be provided or the text revised.**

The data are included in Figure 3H.

**4. Line 816: Fig. 3H legend: "host killing" could lead to confusion since the graph title states "bacterial killing"; the text should be revised (eg, bacterial killing; immune killing)**

Revised.

**5. line 269: should "3.1 days" be 3.5 days,** **per the figure?**

This value is correct because it represents the mean survival time of all the mice in the group. This point is more clearly described in the revised text (page 16, lines 451-452).

**6. S2C and D Fig (lines 32-26): The legend should be improved to clarify what is being shown. Legend to S2C states "Percentage of bacterial clearance in blood circulation of mice post infection as in A", but A does not show percentages, it shows absolute bacterial counts. The legend to panel D is confusing/non-****intuitive perhaps because it describes counting killed bacteria (rather than counting viable bacteria), so the wording could be made clearer to describe what was actually measured and shown.**

Revised.

**7. References:**

**Jensen TS et al, Microbes Infect. 2020 Jan-Feb;22(1):19-30. doi: 10.1016/j.micinf.2019.08.003**

**Spiegelberg HL et al, J Immunol. 1963 May;90:751-9.**

**Brown EJ et al. The role of complement in the localization of pneumococci in the splanchnic reticuloendothelial system during experimental bacteremia. J Immunol. 1981; 126(6): 2230-5.**

Cited in the revise Discussion (page 20, lines 536-540).

**Reviewer #3:**

**1. Line 92 states that how capsule affects pathogenesis is uncharacterized, but the prior paragraph describes several mechanisms by which capsule affects pathogenesis.**

Addressed in revised Introduction (pages 5, lines 92-94).

**2. Line 97: A little more background information about Kupffer cells in the introduction would help readers understand the rationale and impact of the subsequent findings.**

Included in the revised Introduction (page 5, lines 97-108).

**3. Line 111: Please more precisely define** **50% mortality. I think the value used is the time at which half the mice died, but it isn’t clearly stated.**

Revised as “50% mortality within 7 days for the HV strains” (page 6, line 126).

**3. Fig 1C and E; Fig 2C. Is there attrition of sample numbers at 12+ hr? Or does each dot truly represent 3 mice? If there is attrition of sample numbers, how was that accounted for in the statistical analyses?**

These figures represent the data of 3 mice at 12 h although a few of the mice died immediately after retro-orbital bleeding. This information is added to the legends of Fig 1.

**4. Line 164 states that the spleen was the organ with the highest burden of HV Kp, but it appears that sometimes it is the liver is in Fig. 3E.**

We revised this statement (page 11, lines 285-286).

**5. Fig 3H would benefit from plotting the X-axis in scale, as in 3C and 3I.**

Revised (new **Fig 3E**).

**6. Fig 4E and G: the** **Bacteria per FOV – are these absolute values or percentages? Is the bar graph in 4F supposed to be titled “bacteria on KC”? There is a discrepancy between the figure legend and line 228 of the text.**

Bacteria per FOV represent the absolute values of immobilized bacteria detected by IVM imaging. We’ve used the title - “immobilized bacteria” for both the wild type and KC-deficient mice for the quantitative data of IVM (**Fig 5F, 5G**).

**7. Fig 6 – Are naïve mice immunized with vehicle control or no treatment?**

The naïve mice have no treatment. This is defined in the Figure 8 legend.

**8. The methods state that CD1 or C57BL/6 mice were used in the experiments, but the figure legends do not specify which mice are used in which experiments.**

Revised.
